# A developmental evaluation of an intraprofessional **P**ha**r**macy C**om**unication **P**ar**t**nership (PROMPT) to improve transitions in care from hospital to community: A mixed-methods study

**DOI:** 10.1186/s12913-020-4909-0

**Published:** 2020-02-10

**Authors:** Sara J. T. Guilcher, Olavo Fernandes, Miles J. Luke, Gary Wong, Philip Lui, Karen Cameron, Pauline Pariser, Vanessa Raco, Karishma Kak, Shawn Varghese, John Papastergiou, Lisa M. McCarthy

**Affiliations:** 1 Leslie Dan Faculty of Pharmacy, University of Toronto, 144 College Street, Toronto, Ontario M5S 3M2 Canada; 2 Institute of Health Policy, Management and Evaluation, University of Toronto, Toronto, Ontario M5T 3M6 Canada; 3 MAP Centre for Urban Health Solutions, Li Ka Shing Knowledge Institute, St. Michael’s Hospital, 30 Bond Street, Toronto, Ontario M5B 1W8 Canada; 4 University Health Network, 190 Elizabeth Street, Toronto, Ontario M5G 2C4 Canada; 5 Centre for Quality Improvement and Patient Safety, University of Toronto, 2075 Bayview Avenue, Toronto, M4N 3M5 Ontario Canada; 6 Centre for Interprofessional Education, University of Toronto, 399 Bathurst Street, Toronto, M5T 2S8 Ontario Canada; 7 Department of Family and Community Medicine, University of Toronto, Toronto, Ontario Canada; 8 Women’s College Hospital, 76 Grenville Avenue, Toronto, Ontario M5G 1N8 Canada; 9 School of Pharmacy, University of Waterloo, 10A Victoria Street S, Kitchener, ON N2G 1C5 Canada

**Keywords:** Transitions in care, Pharmacists, Medication management, Hospital discharge, Medication reconciliation

## Abstract

**Background:**

People transitioning from hospital- to community-based care are at increased risk of experiencing medication problems that can lead to adverse drug events and poor health outcomes. Community pharmacists provide medication expertise and support during care transitions yet are not routinely included in communications between hospitals and other primary health care providers. The **P**ha**R**macy C**OM**munication **P**ar**T**nership (PROMPT) intervention facilitates medication management by optimizing information sharing between pharmacists across care settings. This developmental evaluation sought to assess the feasibility and acceptability of implementing the PROMPT intervention, and to explore how contextual factors influenced its implementation.

**Methods:**

PROMPT was implemented for 14 weeks (January–April, 2018) in the general internal medicine units at two teaching hospitals in Toronto, Canada. PROMPT featured two contact points between hospital and community pharmacists around patient discharge: (1) faxing an enhanced discharge prescription and discharge summary to a patient’s community pharmacy and (2) a follow-up phone call from the hospital pharmacist to the community pharmacist. Our mixed-method evaluation involved electronic patient records, process measures using tracking forms, telephone surveys and semi-structured interviews with participating community and hospital pharmacists.

**Results:**

The intervention involved 45 patients with communication between 12 hospital and 45 community pharmacists. Overall, the intervention had challenges with feasibility. Issues with fidelity included challenges with the medical discharge summary being available at the time of faxing and hospital pharmacists’ difficulties with incorporating novel elements of the program into their existing practices. However, both community and hospital pharmacists recognized the potential benefits to patient care that PROMPT offered, and both groups proposed recommendations for further improvements. Suggestions included enhancing hospital staffing and resources.

**Conclusion:**

Improving intraprofessional collaboration, through interventions such as PROMPT, positions pharmacists as leaders of medication management services across care settings and has the potential to improve patient care; however, more co-design work is needed to enhance the intervention and its fidelity.

## Background

Improving transitions in care is a key focus for many jurisdictions. People transitioning from hospital- to community-based care are at increased risk of medication therapy problems, which can lead to adverse drug events and poor health outcomes [1–3]. During these vulnerable moments of care transitions, increased collaboration between pharmacists practicing in hospitals and other healthcare providers improves post-discharge outcomes, such as hospital readmissions and emergency department visits [4–6]. Community pharmacists often provide care for patients discharged from hospital; however, they are not routinely included in communications between hospitals and other primary health care providers (e.g., family physicians) [7, 8].

There is growing interest in actively involving community pharmacists as part of interprofessional teams of healthcare providers that help people navigate transitions between hospital and the community [9, 10]. Previous systematic reviews suggest that involving community pharmacists in transitions in care interventions, particularly when they are provided with access to patients’ clinical histories, can increase the identification and resolution of drug therapy problems and reduce drug-related emergency department visits and hospital readmissions [4, 11].

Leveraging the role of community pharmacists for transitions in care and medication management, the **P**ha**r**macy C**om**munication **P**ar**t**nership (PROMPT) intervention was created in 2015 by an interprofessional group of stakeholders in the Greater Toronto Area, Ontario, Canada [12]. Based on best evidence about successful medication management during transitions, the novelty of the PROMPT intervention lies in providing a communication link between pharmacists practicing in hospital and community settings, with the goal of sharing clinically relevant information at discharge to improve care continuity. Unlike many similar pharmacy-led transitions in care interventions [4], the PROMPT intervention was intended to require no additional technology, staffing, or support beyond that already in place in hospital and community pharmacies.

In an earlier evaluation of PROMPT, we focused on describing the characteristics of patients on behalf of whom hospital pharmacists chose to deliver the intervention, the amount and type of communication required between hospital and community pharmacists, and community pharmacists’ preliminary opinions of the intervention [12]. Initial feedback from community pharmacists was positive, yet some provided suggestions on how to further optimize the intervention. For example, some community pharmacists questioned whether a follow-up phone call was necessary, suggesting instead that the contact information of the hospital pharmacist may be sufficient. Notably, our earlier evaluation did not assess the feasibility of the PROMPT intervention, such as whether or not it was delivered with fidelity or if it was acceptable to the hospital pharmacists involved in its delivery. Therefore, our research objectives of the present study were to evaluate hospital and community pharmacists’ perceptions of the feasibility and acceptability of implementing the PROMPT intervention and its’ key components, and to identify and explore how contextual factors influence its implementation.

## Methods

### Study design and setting

This mixed-methods developmental evaluation [13] was conducted between January and April 2018 in the general internal medicine units of two academic hospitals of one health network located in the Greater Toronto Area, Ontario, Canada. The PROMPT intervention is described herein in accordance with the Template for Intervention Description and Replication (TIDieR) Checklist [14]. The protocol was approved by the Institution’s Research Ethics Board.

### Context

#### The PROMPT intervention

**Fig. 1:**
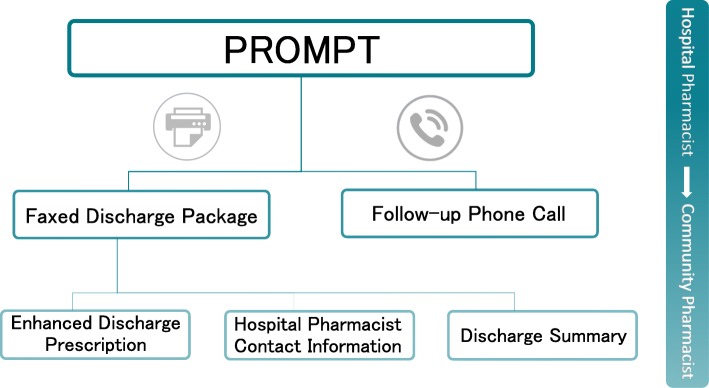
Components of the **P**ha**r**macy C**om**munication **P**ar**t**nership (PROMPT) intervention. The full PROMPT intervention featured two contact points between hospital and community pharmacists around patient discharge: (1) faxing an enhanced discharge prescription and discharge summary to a patient’s community pharmacy and (2) a follow-up phone call from the hospital pharmacist to the community pharmacist

The PROMPT intervention involves outreach by hospital pharmacists to community pharmacists at the time of hospital discharge for eligible patients. The PROMPT intervention (Fig. 1) is composed of two main elements: (1) a discharge package; (2) a follow-up telephone call [12]. First, the hospital pharmacist faxes the discharge package to the community pharmacist. The package includes an enhanced discharge prescription (outlining new medications, those to be continued, and those stopped), a medical discharge summary describing the patient’s course in hospital (e.g., reason for admission, procedures and treatments, diagnostic tests), and the contact information (i.e., name and pager number) of the hospital pharmacist. On the same day, after faxing the discharge package, the hospital pharmacist places a follow-up telephone call to: confirm receipt of the package, determine if the community pharmacist has any questions or concerns and re-emphasize key messages related to continuity of medication therapy. We hypothesized that implementation of the PROMPT intervention would result in improved communication between hospital and community pharmacists.

To understand which components of the PROMPT intervention are most critical to its success, two configurations of the intervention were included in the study protocol based on initial feedback from community pharmacists: full interventions and partial interventions. The full intervention included both the faxed discharge package and the follow-up telephone call. The partial intervention did not require the hospital pharmacist to actively follow up via telephone call with the community pharmacist, although their contact information was still included in the faxed discharge package.

### Study population

Patients were eligible for PROMPT if they were aged 18 years and over, taking five or more medications upon admission to hospital, and had a length of hospital stay greater than 72 h. The minimum 72 h length of stay was chosen to ensure that the hospital pharmacist had adequate time to be involved in the patient’s care. Patients were excluded if they: (1) did not reside in the community (i.e., those admitted from long-term care facilities or other institutions), (2) died during index stay, (3) left the hospital against medical advice, or (4) were transferred to another institution.

A convenience sample of hospital pharmacists was selected to implement the PROMPT intervention. These hospital pharmacists were practicing in the general internal medicine units of two academic hospitals and were registered pharmacists in the province of Ontario, Canada. Prior to implementation of PROMPT, participating hospital pharmacists received training on the intervention components and the processes involved in data collection. A convenience sample of community pharmacists who were recipients of the intervention were recruited over a period of approximately four months.

### Quantitative data collection

We collected quantitative data relating to three main categories of outcomes: (1) patient characteristics, (2) PROMPT process metrics, and (3) community pharmacists’ experiences with PROMPT. Data were collected through electronic hospital medical record audits, PROMPT intervention tracking forms completed by hospital pharmacists, and telephone surveys with community pharmacists.

#### Patient characteristics

A research assistant audited the hospital electronic medical records of study patients to gather key data, including age, sex, number of medications at admission and discharge, and reason(s) for admission.

#### PROMPT process metrics

Hospital pharmacists were provided with both electronic and hardcopy versions of the PROMPT intervention tracking form prior to the data collection period. Hospital pharmacists sent the completed form by email to the study coordinator after delivering the PROMPT intervention to a patient. The tracking form contained the name of the patient, their medical record number, the components of the PROMPT intervention provided to the community pharmacy, contact information for the community pharmacy and name of the community pharmacist (if known). The tracking form also included information regarding unplanned phone calls from the community pharmacist to the hospital pharmacist and the reasons for these calls.

#### Community pharmacist experiences

Within two weeks of a patient's hospital discharge, a member of the PROMPT research team contacted the community pharmacies that had received a PROMPT intervention and asked the pharmacist involved in the patient’s care to complete a telephone survey about their experience with the intervention (questionnaire provided as Additional file 1). Community pharmacists who consented to participate in the survey were asked about the completion of PROMPT components and their opinions regarding the importance and usefulness of these components. The survey contained different types of response options, including binary, ordinal, and open-ended. Three 9-point Likert-style survey questions assessed the importance to patient care of different components of the PROMPT intervention from the community pharmacists’ perspectives (1 = ‘not important at all’; 9 = ‘extremely important’).

### Qualitative data collection

#### Qualitative interviews

Semi-structured qualitative interviews were used to explore hospital and community pharmacists’ perceptions of the PROMPT intervention as well as factors that enhanced or impeded the intervention’s implementation. Upon conclusion of the patient enrollment period (January–April 2018), all hospital pharmacists who had delivered the PROMPT intervention were recruited to participate in telephone qualitative interviews through an email from the study coordinator. A second recruitment email was sent one week later if a reply had not been received. Community pharmacists were recruited to participate in qualitative interviews during the post-intervention telephone survey. Those community pharmacists who expressed interest (*n* = 21) were later contacted for interviews. On completion of the interview, participants received a gift card ($50 CAD).

Interview questions were informed by the Consolidated Framework for Intervention Research (CFIR) [15] and addressed overall experiences with PROMPT, past experiences with transitions in care from hospital to home, and factors that may impact the intervention implementation. Telephone interviews were conducted between June and August 2018. The interviewer wrote reflexive notes after each interview to document general impressions, unique circumstances or context about the interview that could affect its interpretation, and lessons that could be applied to future interviews. Interviews continued until all interested pharmacists had participated; the interviewer’s reflexive notes suggested that no new ideas were identified and that data saturation had likely been reached [16]. All interviews were audio recorded, transcribed verbatim and de-identified.

### Data analysis

Descriptive statistics were computed for all variables using IBM SPSS Statistics, version 22. Categorical variables were described with medians and ranges, continuous variables with means and standard deviations, and nominal variables with frequencies and proportions. The International Statistical Classification of Diseases and Related Health Problems, 10th Revision, Canada (ICD-10-CA) [17] was used to categorize enrolled patients’ reasons for admission to hospital.

Analysis of the interview data was an iterative, constant comparative process [18]. An inductive approach was used for descriptive analyses, involving open coding and identifying themes within the data. Four team members independently reviewed three interview transcripts to identify high-level concepts, after which an initial coding manual was created. These team members independently applied the coding manual to an additional two transcripts to test and refine the manual and the coding process. All transcripts were then catalogued and coded by one team member using NVivo 11 qualitative software. Similar codes were grouped together and patterns that were recognized in the data were discussed by the research team to identify core themes.

#### Triangulation of data

Throughout the implementation phase of PROMPT (January – April 2018), weekly Working Group meetings were held with research team members, including investigators at partner institutions and representatives from the hospital sites’ pharmacy staff who were delivering the PROMPT intervention. The meetings provided a forum for the group to discuss concerns relating to intervention design and implementation. Meeting minutes were used to triangulate the interview data. Themes arising from the interview data were reconciled with similar concepts discussed in the weekly meetings as well as the quantitative data to create a more fulsome interpretation of pharmacists’ experiences with PROMPT.

## Results

Twelve pharmacists across the two hospital sites performed 34 full and 11 partial interventions (45 total interventions) between January and April 2018. Forty-five patients with a mean age of 75 years (SD = 18) received the PROMPT intervention. There were slightly more females (*n* = 25/45, 56%) than males (*n* = 20/45, 44%). Patients were admitted with diagnoses that included a diverse range of medical conditions (such as infectious diseases and conditions relating the circulatory or digestive systems) and were discharged with a median of eight prescription medications (IQR = 4). Of the 45 community pharmacies that were contacted after receiving the PROMPT intervention, two did not consent to a telephone survey and one was a temporary pharmacist who could not be reached, leaving 42 eligible community pharmacists who all participated in telephone surveys.

Thirteen community pharmacists and seven hospital pharmacists consented to participate in qualitative interviews. PROMPT Working Group meetings were held on nine occasions during intervention implementation.

### Feasibility of implementing PROMPT

For the majority of PROMPT interventions (*n* = 44/45; 98%), the intervention tracking forms completed by hospital pharmacists indicated that discharge prescriptions were successfully faxed to the community pharmacy (Table 1). For the one discharge prescription that was not faxed, the hospital pharmacist unsuccessfully tried to send the information many times before providing a verbal prescription by phone. The discharge summary was provided in 22% (*n* = 10/45) of faxed discharge packages. Of the 35 discharge summaries that were not faxed, 51% (*n* = 18/35) were not available at the time of the intervention. In the majority of interventions (*n* = 34/45, 76%), hospital pharmacists stated that their contact information was provided on faxed communication. In all of the 34 full interventions, the hospital pharmacist completed a follow-up telephone call to the community pharmacist confirming receipt of faxed documents, asking if the community pharmacist had additional questions, as well as verbally reviewing medication changes and/or reviewing clinical issues regarding the patient’s recent hospital stay.

**Table 1:** Hospital pharmacist responses to intervention tracking form questions

Tracking Form Items	Yes, *n* (%)	No, *n* (%)	Other,* n* (%)
1. Discharge prescription faxed (*n* = 45)	44 (97.8)	1 (2.2)^1^	–
2. Discharge summary faxed (*n* = 45)	10 (22.2)	35 (77.8)	–
3. Hospital pharmacist’s contact information on faxed documents (*n* = 45)	34 (75.6)	9 (20.0)	2 (4.4)^1,2^
4. Follow-up telephone call to the community pharmacist (*n* = 45)	39 (86.7)^3^	6 (13.3)	–
a. Confirm receipt of faxed documents (*n* = 39)	35 (89.7)^3^	4 (10.3)	–
b. Ask if community pharmacist had additional questions (*n* = 39)	24 (61.5)	15 (38.5)^3^	–
c. Hospital pharmacist reviews medication changes (*n* = 39)	25 (64.1)	14 (35.9)^3^	–
d. Hospital pharmacist reviews issues regarding hospital stay (*n* = 39)	13 (33.3)	26 (66.7)^3^	–

^1^Hospital pharmacist provided a verbal prescription because the fax was busy

^2^Response not documented by hospital pharmacist

^3^Five hospital pharmacists delivering a partial intervention called the community pharmacist only to confirm receipt of faxed documents

Several hospital pharmacists at one site expressed concern at pre-launch planning meetings about not calling community pharmacists during partial interventions to confirm receipt of faxed information or to discuss the possibility of procuring uncommon medications for patients. For this reason, the research team left it to the discretion of the pharmacists whether to complete a partial or full intervention. For the purposes of our analyses, interventions that featured the hospital pharmacist calling the community pharmacy only to confirm receipt of faxed documents were considered partial interventions because no additional clinical information was being exchanged. In 45% (*n* = 5/11) of partial interventions, a telephone call was made from the hospital pharmacists to community pharmacist only to confirm receipt of faxed documents.

In qualitative interviews hospital pharmacists reported that that they had challenges with faxing the discharge summary to the community pharmacist because it was not part of their normal workflow. Further, medical discharge summaries were often not complete at the time that hospital pharmacists were faxing the discharge prescription because these documents are typically written by physicians and are not finalized until the point of discharge. Most hospital pharmacists did not think it would be feasible for them to be expected to fax the discharge summary later, when it became available, because there is no mechanism in place that alerts them when it is finalized. Some suggested that hospital pharmacists could write a concise, pharmacy-specific note in lieu of the discharge summary, or alternatively, administrative staff could fax discharge summaries as they became available (data summarized in Table 2).

**Table 2:** Results from Working Group meetings and semi-structured interviews with community (*n* = 13) and hospital pharmacists (*n* = 7)

	Community Pharmacists		Hospital Pharmacists	
	Pros	Cons	Pros	Cons
Components of PROMPT intervention				
Faxed discharge prescription	- Legible and logically formatted, easy to read - Distinguishes continued, adjusted, discontinued, and newly started medications in hospital - Included the indication for each medication	- Discharge prescriptions sometimes do not include a prescription for all medications that the patient is taking - Occasionally, necessary information (e.g. prescriber license numbers, insurance information) missing from discharge prescriptions - Medications that patients were only intended to be administered in hospital were occasionally included in discharge prescriptions	- *(WG) The lead role that hospital pharmacists have in generating or reviewing discharge prescriptions allows them to tailor those prescriptions so that they are easier for community pharmacists to understand and process	- (WG) Preparing discharge prescriptions and getting them approved and signed by physicians before they can be faxed is a time-intensive part of the PROMPT intervention for hospital pharmacists
Faxed discharge summary	- Useful for clarifying issues that were identified in the discharge prescription, which would otherwise necessitate contacting the prescriber - Included lab values and information about follow-up appointments - If receiving discharge summaries was standard practice, community pharmacists would be more’ familiar and adept at extracting useful information from them - Despite receiving more patient information, community pharmacists did not experience challenges in managing, storing, or subsequently accessing this information	- Contained an overwhelming amount of information, much of which was not relevant to the dispensing-focused role of community pharmacy - Did not contain certain information that is relevant to the dispensing-focused role of community pharmacy - Poor presentation and formatting; relevant information difficult to extract from prose - Time-consuming to read in full	- By providing community pharmacists with more patient and hospital information, hospital pharmacists were more confident that interventions made during a patient’s admission would be maintained in the community	- ^(I + WG) Faxing discharge summaries to community pharmacies is a significant departure from hospital pharmacists’ existing workflows - (I + WG) Discharge summaries were often not yet complete at the time that hospital pharmacists were faxing discharge prescriptions to community pharmacies - Extraneous information included in the discharge summary may obfuscate the more relevant information communicated in the discharge prescription and follow-up telephone call - Concerns about maintaining patient privacy given the volume of personal health information transmitted in the discharge summary
Follow-up telephone call	- Opportunity to discuss the patient’s hospitalization and discharge plan with an informed, reliable source within the hospital - Immediate resolution of prescription issues - Effective way to highlight vital information about the hospitalization and any action items required of the community pharmacy - Allowed community pharmacy to receive patient information that was not included in the faxed documents (e.g. language barriers, living situation) - The standardized telephone call was useful for establishing an open line of communication between the hospital and community pharmacy	- A routine follow-up call may not add value to the intervention, given the community pharmacy has access to the discharge summary and the hospital pharmacist’s contact information	- Enabled hospital pharmacists to convey information that was not included in the faxed documents - Allowed the hospital pharmacist to confirm that the community pharmacy had received the faxed documents - (WG) Follow-up telephone calls were usually short, and subsequent follow-up calls were rarely required	- Routine follow-up calls may not be an efficient use of hospital pharmacists’ time, given that the community pharmacist has the ability to contact them if issues are to arise - (WG) The community pharmacist or pharmacy staff member who received the follow-up call was sometimes not the same as the pharmacist or staff member who received or processed the discharge package
Hospital pharmacist contact info	- Immediate, streamlined resolution of prescription issues - By avoiding challenges and delays in communicating with hospital prescribers, community pharmacy workflow remains unimpeded	- Only useful during hospital pharmacist’s working hours - Contact information was sometimes illegible or incorrect - Would prefer a direct telephone line rather than a pager number	- (WG) Hospital pharmacists are more comfortable giving their contact information to community pharmacists caring for complex patients than to the patients themselves	- Concerns about potentially receiving excessive telephone calls from community pharmacy - Providing their contact information to community pharmacies is not an existing component of hospital pharmacists’ workflows
Mode of PROMPT delivery				
Faxing	- Not discussed	- Faxing degraded the resolution and clarity of important medical documents	- Not discussed	- (I + WG) Hospital pharmacists perceived faxing to be unreliable and time-inefficient. - Faxing personal health information to an unknown location within a community pharmacy could violate patient privacy
Timing of PROMPT delivery				
Faxed discharge prescription	- Receiving a faxed discharge prescription ahead of a complex patient’s arrival allowed the pharmacy to prioritize their workflow and to make necessary arrangements for the patient’s care	- Not discussed	- Expedited delivery of discharge prescription to community pharmacy helps ensure hospital pharmacist’s interventions are quickly implemented in the community	- By faxing the patient’s discharge prescription to their community pharmacy ahead of time, hospital pharmacists could eliminate the patient’s choice of pharmacy and flexibility if their plans were to change suddenly
Follow-up telephone call	- Not discussed	- Not enough time to review faxed documents before receiving follow-up call from hospital pharmacist	- Calling shortly after faxing was a strategy used to make the community pharmacy aware that they had received discharge documents and to encourage them to begin processing the prescription	- Community pharmacists sometimes had not reviewed faxed documents despite hospital pharmacists waiting a considerable amount of time before calling
Who delivers PROMPT				
Faxed discharge summary	- Not discussed	- Not discussed	- It may be feasible for hospital pharmacists to generate a community pharmacy-specific note to be faxed in lieu of the discharge summary, were it not yet available	- Hospital pharmacists are unable to facilitate or influence completion of the discharge summary - Ward clerks or other administrative support could be better positioned than hospital pharmacists to fax documents to community pharmacies
Who receives PROMPT				
Eligibility criteria	- Community pharmacists commonly identified polypharmacy as a characteristic of patients who would benefit from the PROMPT intervention, which was a component of the study’s inclusion criteria (≥ 5 medications upon discharge)	- The study’s enrollment criteria did not explicitly consider many other patient characteristics (e.g. psychiatric illness, physical impairment, those who require compliance packaging) that community pharmacists believed would help identify the most appropriate patients for the PROMPT intervention - Community pharmacists identified a broad, heterogeneous inventory of patient groups that they felt would most benefit from PROMPT	- Hospital pharmacists agreed that polypharmacy was a useful criterion for identifying patients who benefit the most from the PROMPT intervention - A large proportion of the patients encountered in the hospital pharmacists’ practices, including most they believed would benefit from the intervention, satisfied the study’s eligibility criteria	- Hospital pharmacists identified certain patient characteristics (e.g. advanced age, many changes made to a patient’s medication regimen while in hospital) that they felt made a patient ‘complex’, but that were not included in the study’s enrollment criteria - (I + WG) Some patients who did not satisfy the length of hospital stay criterion (≥ 72 h) may still have benefitted from the intervention - The length of stay criterion was the most difficult criterion for hospital pharmacists to confirm was satisfied - (WG) There were intervals during the data collection period of the study when only few patients eligible for PROMPT were admitted under general internal medicine - (WG) There were instances where even if a patient satisfied the study’s eligibility criteria they were not enrolled because there were no changes made to the patient’s medications in hospital and, therefore, no prescription was generated to send to the patient’s community pharmacy

*(WG) = idea discussed during PROMPT working group’s weekly meetings

^(I + WG) = idea discussed during working group meetings and during interviews with pharmacists

All other ideas were discussed only during interviews

Preparing and faxing discharge prescriptions and connecting with community pharmacists via telephone were components of PROMPT that some hospital pharmacists reported doing outside of the study, particularly for the complex patients targeted by the study’s eligibility criteria. Faxing discharge summaries and providing their own contact information, conversely, were departures from their typical practices.

### PROMPT acceptability

Overall, despite challenges with fidelity, we found that PROMPT was well received by pharmacists practicing in both hospital and community settings. Hospital and community pharmacists identified key features of the intervention that were acceptable, as well as possible enhancements to improve implementation. In general, community pharmacists highlighted minimal challenges with incorporating PROMPT into their existing practice. The additional medication information presented in the enhanced discharge prescription was reported by community pharmacists as a key benefit, as was the fact that it was faxed to the community pharmacy prior to the patient’s arrival. This allowed pharmacy staff to prioritize their workflow and to make arrangements that facilitated timely and accurate dispensing of all prescribed medications (e.g., blister packaging medications or ordering additional inventory).

*“Patient care-wise, it enables us to prevent the DRPs [drug related problems]. It enables us also to prepare anything, counselling-wise, that needs to be addressed. Any non-pharmacological stuff that’s related to new therapies, we’ll be able to prepare for that also ahead. Also, by having that contact information, anything that will require time, we’ll be able to clarify at the same time - I don’t have to wait until the next day or fax different doctors so that I can get an answer. I’ll be able to basically provide the care that they need efficiently and accurately because it’s also printed, everything is complete, and I’ll be able to ask questions if some stuff don’t make sense to me, the rationale behind certain things that are written on the prescription.”*

- Community Pharmacist, Participant ID #740

Further, community pharmacists recognized that although PROMPT required more time up-front because they received a telephone call from the hospital pharmacist, it saved them time further downstream in the patient care process by providing an opportunity for rapid and direct identification and resolution of prescription issues.

*“I actually quite like this program and taking the time to talk to pharmacists from the hospital. I find that it’s definitely just beneficial, almost no drawbacks … if I didn’t have the telephone call from the hospital to immediately resolve a missing LU [limited use] code or questionable indication or an interaction situation, that actually takes me more time to do the write-up, to go through the clinical or therapeutic process we have. Why is the patient taking those two drugs at such dose, and what’s the reason behind it?”*

- Community Pharmacist, Participant ID #985

A primary concern expressed by community pharmacists was the uncertainty about their ability to devote as much attention as they would like to each intervention, specifically reviewing the discharge summary for every patient (unless there were prescription issues that could potentially be resolved using the information contained in it). Some community pharmacists reported that the faxed discharge summary contained an overwhelming amount of information, much of which was not related to the specific aspects of patient care that involve community pharmacists. However, other community pharmacists felt that the discharge summaries provided them with a more holistic understanding of their patient and the potential to enhance the provision of care. While community pharmacists may not be able to read every patient’s faxed discharge summary in depth, they appreciated the sharing of this information. They felt it would save them time and effort by providing answers to clinical questions and prescription issues that, if they were to arise, would have otherwise created a need for the pharmacist to contact the hospital for clarification. Moreover, community pharmacists had concerns about the quality of the faxed information, with faxing being described as a less than ideal mode to transmit information due to its propensity to degrade the resolution and clarity of the documents.

Incorporating the follow-up telephone call as a routine component of PROMPT was questioned by some community pharmacists who felt that receipt of the hospital pharmacist’s contact information was adequate. However, a common comment was that the contact information would only be useful as long as the pharmacist was accessible at the hospital. Community pharmacies have longer hours of operation than hospital pharmacists, resulting in a lack of after-hours support.

*“ … I know the pharmacist will provide their information in case we need to reach them, which is great. Like, I guess for normal business hours. But hypothetically, what if I needed to contact them and they’re no longer there. Again, I would still have to wait and the patient would have to wait.”*

- Community Pharmacist, Participant ID #140

Hospital pharmacists reported more heterogeneous opinions about the acceptability of the PROMPT intervention. They recognized the benefits that their community counterparts received when they included their contact information on the discharge prescription; however, they expressed some hesitation in integrating this approach as a standard practice. They were concerned that they would begin to receive more calls from community pharmacies than they could manage, and that many of those calls would be unrelated to the admission in which they had been involved.

*“we’re a little bit protective about how many people end up with our phone numbers because otherwise you get calls six months down the road asking for refills for something that’s not appropriate. But I think as long as it came with a caveat of that we try to write on there that it was like two days or five days or whatever, as long as that also auto populated, I think it would be acceptable.”*

- Hospital Pharmacist, Participant #B-32

Some hospital pharmacists also questioned whether community pharmacists were in patients’ circles of care, and as such whether they were able to receive patients’ personal health information without patient consent. Similarly, hospital pharmacists were also concerned about the potential for compromised security of their patients’ personal health information after faxing it to the community pharmacy.

*“There’s a lot of information in the discharge summary and you know, the idea that this gets faxed to a fax machine in a whole pharmacy where anyone could access it and they’re not used to getting this information. So, are they going to store or dispose of this properly? Or, you know, is the patient’s information going to be everywhere?”*

- Hospital Pharmacist, Participant #A-2

Most of the hospital pharmacists also expressed a desire for an alternative method to faxing for sending documents to community pharmacies. For hospital pharmacists, this desire was due to the unreliability of faxing and inability to confirm receipt of faxed documents, and because it requires time that could be better spent performing other clinical duties.

*“Sometimes the fax machine was busy, someone was using it, it just takes some time, extra couple of minutes added. I think there would have been one instance, that the pharmacies, once they’re called to confirm receipt of the prescription, they didn’t actually get it and that’s unfortunately not uncommon for faxes that we think they go through and they say everything is fine on our side but then we call the pharmacy and for whatever reason, it didn’t make it to them. So, that’s a little bit of a hurdle with using kind of the fax system.”*

- Hospital Pharmacist, Participant #B-12

#### Importance of PROMPT components

Community pharmacists considered the faxed discharge prescription with the contact information of the hospital pharmacist to be important, with a median score of 9 (IQR = 0) (Table 3). Community pharmacists also considered the faxed discharge summary to be important in providing care, although Likert-type scores were more heterogeneous (median = 8, IQR = 3). The community pharmacists participating in the full intervention scored the follow-up telephone call from the hospital pharmacist slightly lower (median = 8, IQR = 2) than the community pharmacists in the partial intervention (media*n* = 9, IQR = 2), however both rated the follow-up call to be important (Table 3).

**Table 3:** Community pharmacists’ (*n* = 42) rating of importance of the various PROMPT components*

Survey Questions	Median (IQR)
How important is the faxed prescription with the contact information of the hospital pharmacist?	
Full^a^	9 (1)
Partial^b^	9 (0)
All Respondents	9 (0)
How important is the Faxed Discharge Summary?	
Full	8 (2)
Partial	8 (3)
All Respondents	8 (3)
How important is a direct telephone call from the hospital pharmacist involved in the patient’s care?	
Full	8 (2)
Partial	9 (2)
All Respondents	9 (2)

*Scale 1 to 9; 1 = not at all important, 9 = extremely important

^a^ Sample size for full was 31

^b^ Sample size for partial was 11

#### Perceptions of PROMPT on patient care

Community pharmacists were asked if they experienced or anticipated any issues in providing care or in dispensing the medications that their patients had been prescribed at discharge. Nearly half of the respondents who had received the full intervention (n = 13/30, 43%) had concerns with providing care for their patient. For those participating in the partial intervention (*n* = 11), three (27%) had concerns providing care for their patient. Of these three respondents, two followed-up with the hospital pharmacist by telephone. The types and frequencies of community pharmacists’ concerns are presented in Table 4.

**Table 4:** Community pharmacists’ (*n* = 16) concerns in providing care to patients enrolled in PROMPT

Concern in providing care for PROMPT patient	N*
Incomplete discharge instructions	5
Incorrect/query dosage	5
Money/financial barriers	2
Duplication of therapy	1
No caregiver and needs assistance	1
Too many doctors and/or pharmacies involved in the patient’s care	1
Community pharmacist requesting the hospital provide one day supply of medication because the discharge occurred after the community pharmacy’s hours of delivery	1

* A total of 16 community pharmacists identified concerns in providing patient care

Of the 15 survey respondents who spoke with a hospital pharmacist about their concerns in caring for their patient, 13 reported a resolution of their concerns. In some of the interventions (*n* = 9/45; 20%), the community pharmacist initiated an unplanned follow-up telephone call with the hospital pharmacist. Of the nine community pharmacist-initiated follow-up telephone calls, proportionately more calls were made by community pharmacists who received the partial intervention (*n* = 3/11, 27%) than by those who received the full intervention (*n* = 6/34, 18%). The majority of telephone calls (*n* = 8/9, 89%) were made to clarify the discharge prescription; specifically, to resolve issues regarding dose, frequency, formulation, duration of therapy, timing of medication administration or reassessment of medications stopped or held in hospital. One follow-up telephone call was made to clarify an administrative issue (i.e., a missing Limited Use code).

### Implementation considerations for PROMPT

Participants identified several factors that influenced the implementation of PROMPT, which included: initial unfamiliarity with PROMPT; existing hospital workflow; staffing, competing priorities, and rushed discharges; and organizational buy-in. Each of these factors will be discussed in more detail below.

#### Community pharmacists’ initial unfamiliarity with PROMPT

Community pharmacists described their initial unfamiliarity with the PROMPT intervention, which led to sometimes not knowing what to do with the information they had received, as well as where to find the relevant pieces of information they needed in the faxed documents. For example, community pharmacists described not knowing where to find pertinent information within the discharge summary they were sent.

*“It was challenging just in that when they were going to fax it to me, it was something new – it was not standard for me, it’s a lot of paper … But also, if it becomes standard with all the hospitals then all of us would be more alert and if they would have a standardized form then my eyes will get used to, okay, how did this paper look like and where the information is, as I read through it I’ll be able to focus on the information that I need right now on the spot when the hospital pharmacist is on the line.”*

- Community Pharmacist, Participant #740

#### Existing hospital workflow

When asked why several PROMPT interventions did not feature inclusion of the hospital pharmacist’s contact information on the discharge prescription, hospital pharmacists reported forgetting to include it because, much like faxing discharge summaries, doing so is not a part of their normal practice. Some hospital pharmacists also reported providing their contact information to the community pharmacy over the phone (rather than written on the discharge prescription), and therefore not documenting it on intervention tracking forms. Suggestions for overcoming these barriers included: giving hospital pharmacists a checklist outlining each step in the PROMPT process; sending email reminders in the weekly project update circulated by study personnel; generating a standardized fax cover page that designated space for contact information for communications from hospital to community pharmacy; or adding a designated space for the pharmacist’s contact information on the discharge prescription or the discharge reconciliation software used by the hospital.

#### Staffing, competing priorities and sudden discharges

Staffing levels were sometimes inadequate to comprehensively manage all of the patients admitted to internal medicine. As such, there were times when hospital pharmacists described challenges with performing tasks beyond their core functions of medication distribution and reviewing medication orders for safety. When this was the case, PROMPT became a lower priority and was not done as frequently.

*“Yeah, I think staffing is huge! … in terms of how many people we’re caring for at the time, because as that number goes up – as the number I’m responsible for goes up, my ability to do things like PROMPT goes down.”*

- Hospital Pharmacist, Participant ID #B-32

An additional factor identified by hospital pharmacists was that patients sometimes were discharged suddenly without the knowledge or involvement of the pharmacy team. In these cases, hospital pharmacists could not complete the various components of the PROMPT intervention (e.g., send the discharge summary).

#### “Buy-in” at organizational level

Some hospital pharmacists shared a perception of institutional emphasis on admission medication reconciliations over discharge care. These hospital pharmacists suggested that an increased organizational emphasis from senior leadership on pharmacy’s involvement at discharge would facilitate the incorporation of PROMPT into their practice.

*“And again, if there were actually enough resources and leadership support to say, ‘Pharmacists’ involvement in discharge is expected, as their involvement in admission is’.”*

*-* Hospital Pharmacist, Participant ID #A-2

## Discussion

This study sought to explore the feasibility, acceptability and context influencing the implementation of the PROMPT intervention. Understanding how PROMPT was delivered informs the resources required for adaptation and spread to additional contexts. Determining the critical components of PROMPT is necessary to ensure that the intervention, when spread to other institutions, will produce similar impacts on hospital processes and patient care. Many complex/multi-component pharmacy-led transitions in care interventions have been evaluated in the literature in recent years; however, very few have evaluated intervention fidelity [5, 6, 19–21]. Overall, we found that the PROMPT intervention was delivered with sub-optimal fidelity for a variety of reasons, including the medical discharge summary being unavailable at the time of faxing and hospital pharmacists’ difficulties in fully incorporating novel elements of the intervention into their existing practices. However, both community and hospital pharmacists recognized the potential benefits to patient care that PROMPT offered, and both groups proposed recommendations for further optimizing the intervention. Additional staffing and resources could better equip hospital pharmacists to prioritize the key elements of PROMPT.

Overall, the PROMPT intervention had sub-optimal fidelity largely due to issues concerning the full versus partial intervention options and minimal control over some of the components (e.g., discharge summary availability). To further explore findings from our previous evaluation of PROMPT [12] about the value of a routine follow-up telephone call between the hospital and community pharmacists, the current study’s protocol proposed an analysis of pharmacists’ perceptions of full versus partial interventions. However, in meetings with hospital pharmacists prior to launch of the study, pharmacists at one site opted to not perform partial interventions because they felt this variation of the intervention may restrict their ability to deliver comprehensive patient care. For this reason, it was left to the discretion of the hospital pharmacists to determine the appropriate components of the intervention to be delivered to each patient enrolled in the study. This impacted the study’s ability to explore which components of the PROMPT intervention are integral to its success and how it can be further optimized.

The approach of using an active follow-up phone call has been previously used in a recent study led by Ravn-Nielsen and colleagues [6]. The OPTIMIST trial included a follow-up telephone call from the hospital pharmacist to the community pharmacist only if the hospital pharmacist considered it necessary [6]. While this component of the intervention was not evaluated in isolation, overall results from the trial demonstrated lower 30- and 180-day hospital readmission rates for patients who received extended pharmacist intervention. An economic evaluation of the intervention showed that costs incurred due to increased pharmacist follow up were offset by reduced readmission-related costs [22].

Using CFIR [15] as a sensitizing framework when developing our interview guides was helpful for probing salient concepts and perspectives that would otherwise have been left unexplored. For example, interviews highlighted important perceived gaps in organizational support, such as how hospital pharmacists were encouraged to participate in the study yet were also managing multiple competing patient care priorities. Some hospital pharmacists perceived that there was organizational emphasis for pharmacists to prioritize providing patient care at the time of admission, whereas discharge-focused care is only prioritized for selected highest risk patients. This perception may arise partly from medication reconciliation responsibilities of hospital pharmacists within the organization and the recognition that if medication reconciliation on admission is poorly done, accuracy of medication reconciliation at discharge cannot be achieved.

Hospital pharmacists’ ability to enroll patients and deliver the PROMPT intervention to community pharmacists seemed to be challenged particularly during periods of limited resources with short-staffing relative to high patient demands. With health jurisdictions placing more emphasis on integrated care and improved care transitions from hospital to home [23], our findings reinforce the need to align priorities with organizational support and appropriate resources (e.g., more pharmacists on staff and administrative support for sharing information to community team members, such as community pharmacists). Similarly to the OPTIMIST trial, Wright et al. (2019) demonstrated that increased involvement of pharmacists in the care of patients with complexity across transitions has the potential to reduce healthcare costs by improving rates of hospital readmissions [5], providing strong rationale for reallocating resources to enable pharmacists to play greater roles in discharge and transitional care.

Opportunities for future research include continuing to refine PROMPT and evaluate the implementation in different settings beyond urban teaching hospitals. Particular refinements would include exploring how other communications technology (e.g. secure electronic messaging systems between hospitals and primary care, rather than facsimile) can be leveraged to enhance the efficiency and reliability of PROMPT. Further, increasing resources to offset administrative tasks of hospital pharmacists may enhance uptake of the intervention. Finally, involving physicians within the PROMPT intervention more formally may improve interprofessional collaboration and the timeliness and content within a discharge summary. Our findings support the need for more research to understand what are the optimal content and format of a discharge summary that is usable for pharmacists, patients and physicians.

### Limitations

Our intended comparative analysis of full versus partial interventions aimed to explore the different components of the PROMPT intervention based on previous feedback, but, as discussed, this did not occur when hospital pharmacists at one site agreed only to deliver full interventions. This is both a limitation of this study as well as a valuable learning regarding the acceptability of partial interventions within competing hospital and community pharmacists’ priorities. Future research would be warranted to explore perceptions of full versus partial interventions in other settings beyond our study hospital sites and design tailored interventions.

Another limitation of the study was concerns about the eligibility criteria, such as patients having to have spent at least 72 h in hospital, as some hospital pharmacists found this was difficult for them to verify. If a patient was transferred to their care from a different unit within the hospital then they were often unsure exactly when they had been admitted to hospital, and therefore did not enroll the patient. Additionally, many hospital pharmacists felt that this criterion was restrictive as they reported caring for complex patients who they felt would benefit from PROMPT, but who were ineligible for enrollment because they were in hospital for fewer than 72 h. Moreover, further research would be warranted in determining what patients would benefits the most from PROMPT (beyond five or more medications) and how to best to determine high risk.

## Conclusions

Transitions in care, including from hospital to home, are vulnerable moments for patients that place them at risk of experiencing adverse drug events [1–3]. The PROMPT intervention aims to mitigate this risk by facilitating communication between hospital and community pharmacists. While some hospital pharmacists viewed PROMPT as an addition to their workload, many welcomed it as an opportunity to extend their roles as healthcare providers to ensure that the focused care that their patients received in hospital was being sustained through the transition back into the community. Despite sub-optimal intervention fidelity, community pharmacists reported favourable opinions of the intervention, which improved information sharing, streamlined workflows, and enhanced ability to provide care for complex patients. Additional hospital resources for discharge planning are critical for future wide scale implementation.

Ultimately, PROMPT has the potential to improve patients’ medication experiences, safety, integration of care, overall health and quality of life while reducing health-system costs. The model is practical, generalizable and strengthens the position of pharmacists as leaders of medication management services across care settings; however, more co-design work is needed to enhance the intervention and its fidelity.

## Supplementary information

**Additional file 1.** Brief Community Pharmacist Survey – Full Intervention.

## Data Availability

The datasets generated and/or analyzed during the current study are not publicly available due to inclusion of many direct and indirect participant identifiers, but de-identified information is available from the corresponding author on reasonable request.

## Abbreviations

CFIR: Consolidated Framework for Implementation Research
ICD-10-CA: International Statistical Classification of Diseases and Related Health Problems, 10th Revision, Canada
PROMPT: **P**ha**r**macy C**om**munication **P**ar**t**nership
TIDieR: Template for Intervention Description and Replication
