# Supplementary material for: A developmental evaluation of an intraprofessional Pharmacy Comunication Partnership (PROMPT) to improve transitions in care from hospital to community: A mixed-methods study
Source: BMC Health Serv Res. 2020 Feb 10;20:99. doi: 10.1186/s12913-020-4909-0 (PMC7011369; doi:10.1186/s12913-020-4909-0)
Supplement: Supplementary file 1 — Additional file 1. Brief Community Pharmacist Survey – Full Intervention. [file 12913_2020_4909_MOESM1_ESM.docx]

**Brief Community Pharmacist Survey – Full Intervention**

Name of Pharmacist _______________________________________________

Name of Pharmacy _______________________________________________

1. Did [*patient name*] contact your pharmacy to about their prescriptions following discharge from the hospital [*insert date*]?

Yes

No

Do not know/Cannot recall

1. Did you receive the faxed information from the UHN pharmacist about [*patient name*]’s recent discharge from the hospital?

Yes

No

Do not know/Cannot recall

1. In the faxed information, did you receive a faxed prescription for [*patient name*]?

Yes

No

Do not know/Cannot recall

1. In the prescription, did you notice the pharmacist’s contact name and pager number?

Yes

No

Do not know/Cannot recall

1. Did you fill the prescription for [*patient name*]?

Yes

No

Do not know/Cannot recall

If no, why not?

1. In the faxed information, did you receive a copy of the discharge summary? The discharge summary includes detailed information on the reason for admission, tests/procedures during admission, length of hospital stay, and discharge plan.

Yes

No

Do not know/Cannot recall

If no, skip to question #8

1. If yes, was the information in the discharge summary useful to you?
   1. If yes, how was it useful?
   2. If no, why not useful?
2. Did you receive a phone call from the UHN pharmacist related to [*patient name*]?

Yes

No

Do not know/Cannot recall

If no skip to question #9

1. Did you speak with the hospital pharmacist when he/she first called?

Yes

No

- 1. If no, did you eventually connect with the pharmacist at a later time?

Yes

No

1. Did you have any concerns or issues with the providing care for [*patient name*]?

Yes

No

Do not know/Cannot recall

1. If yes, what were the concerns/issues: (open-ended)

Adverse drug reaction or side effect

Money/financial barriers

Prescribed with known allergy/intolerance

Discharge instructions incomplete/inaccurate/illegible

Duplication

Incorrect label

Incorrect dosage

Incorrect quantity

Cognitive impairment not recognized

No caregiver and needs assistance

If no, skip to #12

1. Were your concerns resolved over the phone with the UHN pharmacists?

Yes

No

Do not know/Cannot recall

1. If no, why not?
2. On a scale of 1-9, with 1 being not important to 9 being extremely important, how important would the following types of communication be to you in providing care for patients who have been recently discharge from the hospital?
   1. Faxed prescription with the contact info of the acute hospital pharmacist

| 1 | 2 | 3 | 4 | 5 | 6 | 7 | 8 | 9 |
| --- | --- | --- | --- | --- | --- | --- | --- | --- |
| Not Important |  |  |  |  |  |  |  | Extremely Important |

- 1. Faxed discharge summary

| 1 | 2 | 3 | 4 | 5 | 6 | 7 | 8 | 9 |
| --- | --- | --- | --- | --- | --- | --- | --- | --- |
| Not Important |  |  |  |  |  |  |  | Extremely Important |

- 1. Direct phone call from acute hospital pharmacist involved in the patient’s care

| 1 | 2 | 3 | 4 | 5 | 6 | 7 | 8 | 9 |
| --- | --- | --- | --- | --- | --- | --- | --- | --- |
| Not Important |  |  |  |  |  |  |  | Extremely Important |

- 1. Other [list]

1. Have you recently received a discharge package from UHN for another patient (i.e., within the last few months)?

Yes

No

Do not know/Cannot recall

**Brief Community Pharmacist Survey – Partial Intervention**

Name of Pharmacist ______

Name of Pharmacy ______

1. Did [*patient name*] contact your pharmacy to about their prescriptions following discharge from the hospital [*insert date*]?

Yes

No

Do not know/Cannot recall

1. Did you receive the faxed information from the UHN pharmacist about [*patient name*]’s recent discharge from the hospital?

Yes

No

Do not know/Cannot recall

1. In the faxed information, did you receive a faxed prescription for [*patient name*]?

Yes

No

Do not know/Cannot recall

1. In the prescription, did you notice the pharmacist’s contact name and pager number?

Yes

No

Do not know/Cannot recall

1. Did you fill the prescription for [*patient name*]?

Yes

No

Do not know/Cannot recall

1. If no, why not?
2. In the faxed information, did you receive a copy of the discharge summary? The discharge summary includes detailed information on the reason for admission, tests/procedures during admission, length of hospital stay, and discharge plan.

Yes

No

Do not know/Cannot recall

If no, skip to question #8

1. If yes, was the information in the discharge summary useful to you?

Yes

No

Do not know/Cannot recall

- 1. If yes, how was it useful?
  2. If no, why not useful?

1. Did you have any concerns or issues with providing care for [*patient name*]?

Yes

No

Do not know/Cannot recall

If yes, what were the concerns/issues: [open ended]

Adverse drug reaction or side effect

Money/financial barriers

Prescribed with known allergy/intolerance

Discharge instructions incomplete/inaccurate/illegible

Duplication

Incorrect label

Incorrect dosage

Incorrect quantity

Cognitive impairment not recognized

No caregiver and needs assistance

1. Did you phone the UHN pharmacist for any of these concerns or follow up questions?

Yes

No

Do not know/Cannot recall

If no, skip to #12

1. Did you speak with the hospital pharmacist when you first called?

Yes

No

- 1. If no, did you connect with the pharmacist at a later time?

Yes

No

If no, go to # 12

1. Were your concerns resolved over the phone with the UHN pharmacists?

Yes

No

1. If no, why not?
2. On a scale of 1-9, with 1 being not important to 9 being extremely important, how important would the following types of communication be to you in providing care for patients who have been recently discharge from the hospital?
   1. Faxed prescription with the contact info of the acute hospital pharmacist

| 1 | 2 | 3 | 4 | 5 | 6 | 7 | 8 | 9 |
| --- | --- | --- | --- | --- | --- | --- | --- | --- |
| Not Important |  |  |  |  |  |  |  | Extremely Important |

- 1. Faxed discharge summary

| 1 | 2 | 3 | 4 | 5 | 6 | 7 | 8 | 9 |
| --- | --- | --- | --- | --- | --- | --- | --- | --- |
| Not Important |  |  |  |  |  |  |  | Extremely Important |

- 1. Direct phone call from acute hospital pharmacist involved in the patient’s care

| 1 | 2 | 3 | 4 | 5 | 6 | 7 | 8 | 9 |
| --- | --- | --- | --- | --- | --- | --- | --- | --- |
| Not Important |  |  |  |  |  |  |  | Extremely Important |

- 1. Other [list]

1. Have you recently received a discharge package from UHN for another patient (i.e., within the last few months)?

Yes

No

Do not know/Cannot recall
